# A cross-organizational Lean deployment in an Italian regional healthcare system

**DOI:** 10.1108/IJHCQA-06-2023-0045

**Published:** 2023-11-16

**Authors:** Angelo Rosa, Giuliano Marolla, Olivia McDermott

**Affiliations:** Department of Management, Finance and Technology, Universita LUM Giuseppe Degennaro, Casamassima, Italy; College of Science and Engineering, University of Galway, Galway, Ireland

**Keywords:** Lean healthcare, Organizational models, Critical success and failure factors, Longitudinal analysis, Lean adoption phases

## Abstract

**Purpose:**

This study explores how Lean was deployed in several hospitals in the Apulia region in Italy over 3.5 years.

**Design/methodology/approach:**

An exploratory qualitative design was drawn up based on semi-structured interviews.

**Findings:**

The drivers of Lean in hospitals were to increase patient satisfaction and improve workplace well-being by eliminating non-value-add waste. The participants highlighted three key elements of the pivotal implementation stages of Lean: introduction, spontaneous and informal dissemination and strategic level implementation and highlighted critical success and failure factors that emerged for each of these stages. During the introduction, training and coaching from an external consultant were among the most impactful factors in the success of pilot projects, while time constraints and the adoption of process analysis tools were the main barriers to implementation. The experiences of the Lean teams strongly influence the process of spontaneous dissemination aided by the celebration of project results and the commitment of the departmental hospital heads.

**Practical implications:**

Lean culture can spread to allow many projects be conducted spontaneously, but the Lean paradigm can struggle to be adopted strategically. Lean in healthcare can fail because of the lack of alignment of Lean with leadership in healthcare and with their strategic vision, a lack of employees' project management skills and crucially the absence of a Lean steering committee.

**Originality/value:**

The absence of managerial expertise and a will to support Lean implementation do not allow for systemic adoption of Lean. This is one of the first and largest long-term case studies on a Lean cross-regional multi-hospital application in healthcare.

## 1. Introduction

Continuously rising healthcare costs and increased patient populations requiring healthcare, as well as high variability in operational processes, have required national and local healthcare organizations to explore new ways to increase the levels of patient service quality and to add value for stakeholders (McDermott *et al.*, 2021; Rosa *et al.*, 2021). Lean management has been recognized as one of the most effective operational excellence methodologies to improve operational performance and the reduction of waste (Graban, 2016). Interest in Lean in healthcare has grown among researchers and practitioners in recent years (Al-Balushi *et al.*, 2014; McDermott *et al.*, 2022a). Many examples of Lean hospital applications have been published (Ahmed *et al.*, 2018; Rosa *et al.*, 2023a). Although many articles discuss performance improvements in hospitals or healthcare services arising from Lean adoption, many focus only on stand-alone case studies or individual process improvements but not systemic implementations across the entire organization (Radnor *et al.*, 2011; de Souza and Pidd, 2011). The literature demonstrates a lack of skill in managing the critical failure factors many organizations experience when implementing Lean at the systemic level (Al-Balushi *et al.*, 2014; McDermott *et al.*, 2022a). Many examples of Lean implementation that are “guided by principles” are rare (Marolla *et al.*, 2022; Antony *et al.*, 2018). Developing the skills needed to implement Lean at a systems level requires strong organizational and managerial support from healthcare organizations (Al-Balushi *et al.*, 2014; Radnor *et al.*, 2011). Some healthcare organizations cannot undertake a Lean deployment effectively owing to efforts due to structural or organizational constraints and therefore need external support to make them effective (Bhat *et al.*, 2019; McDermott *et al.*, 2022). In this situation, healthcare government agencies can play a critical role by delivering programs for introducing and deploying Lean to assist them in its adoption by healthcare organizations (Antony *et al.*, 2021; White, 2018). While some studies demonstrate the role of healthcare agencies in deploying Lean, many do not investigate how the deployment of Lean is experienced and the long-term results (McDermott *et al.*, 2022a; Robert *et al.*, 2020). There is a gap in the literature in relation to how Lean implementation programs are promoted by healthcare agencies and how they facilitate the adoption of the paradigm is still unexplored.

This study discusses the experiences of several Italian hospitals involved in the Lean introduction and deployment program “Lean Lab” – promoted by the Italian Apulia Regional Strategic Agency for Health and Social Care (AReSS). The study aims to explore issues related to Lean implementation in multiple healthcare organizations and aims to answer the following questions:

RQ1. What factors drove the regional initiative's success or failure?

RQ2. What are the participants' experiences in Lean introduction and dissemination in their organizations?

RQ3. How have hospitals managed the sustainability of Lean implementation over time?

To assess the effectiveness of the Lean strategic introduction and dissemination program, a qualitative research methodology was used with semi-structured interviews over a large time period of 3.5 years. Section 2 discusses the literature review, Section 3 the research methodology, Section 4 the results and Sections 5 and 6 the discussion and conclusion.

A preliminary version of this work has been reported in “Implementation Experiences of Lean Organization in Healthcare for Apulian Hospitals: A Longitudinal Interview In-Depth Study” Lean, Green and Sustainability. ELEC 2022. International Federation for Information Processing (IFIP) Advances in Information and Communication Technology, vol 668. Springer, Cham. https://doi.org/10.1007/978-3-031-25741-4_5.

## 2. Literature review

Lean is an operational excellence paradigm integrating various approaches and methods focused on waste reduction, employee empowerment and continuous process improvement (Womack and Jones, 1996). Over the past 2 decades, the healthcare sector's adoption rate has grown very high (Chiarini and Bracci, 2013; Rosa *et al.*, 2021). Unlike other managerial methodologies aimed at process improvements, the choice of Lean implementation by healthcare organizations has proven to be not a fad but a growing trend (Marolla *et al.*, 2022; McDermott *et al.*, 2022b). The great interest of healthcare organizations in this paradigm is due to the numerous testimonies of the benefits achieved in patient pathways support processes and related to the organization's work environment (Ahmed *et al.*, 2018; Rosa *et al.*, 2023b; Trakulsunti *et al.*, 2021). The lead and waiting times reduction and the quality and appropriateness of care improvements are just some benefits that Lean projects can achieve. Implementing Lean in healthcare processes has been shown to improve risk and patient safety performance, promote learning across multiple disciplines, enhance vertical and horizontal organizational communication, and facilitate the creation and adoption of standard work (Bhat *et al.*, 2019; Trakulsunti *et al.*, 2021). Many improvements utilizing Lean are related to supporting processes, including increasing resource availability, for example, in operating theatres and diagnostic laboratories, reducing waste from transportation (e.g. drug movements, space utilization and layout), and reducing over-processing (e.g. referral activities, discharge administrative activities (McDermott *et al.*, 2022b). Many authors argue that the paradigm is particularly effective in the healthcare sector because physicians and healthcare professionals easily understand its concepts and tools as they are already geared toward creating value for patients and using tools to control and standardize clinical processes (Joosten *et al.*, 2009; Radnor *et al.*, 2011). Although the methodology is increasing in popularity in seems to be increasingly popular in the health sector, in most cases, it is merely applied at the level of stand-alone clinical or support processes and not across organizations (Al-Balushi *et al.*, 2014; Antony *et al.*, 2021; McDermott *et al.*, 2022b). This micro-level implementation is typical of organizations that are introducing the Lean paradigm or has a low level of maturity in their deployment (McDermott *et al.*, 2022a; de Souza and Pidd, 2011). Organizations that want to exploit Lean's full value must disseminate and adopt its concepts at all levels and apply them systemically (Antony *et al.*, 2021; Kaplan *et al.*, 2012). Systemic-level implementation characteristics include a continuous improvement culture deeply embedded within the organization (Rosa *et al.*, 2021).

To achieve systematic levels of implementation, there must be team-based decision-making systems, the use of improvement-oriented management systems aligned with key strategies, a high staff maturity in the use of Lean tools, regular review of improvement programs, formalized Lean management systems and alignment between strategic and operational Lean objectives (McDermott *et al.*, 2021; McIntosh *et al.*, 2014). The most recent empirical studies have shown that when Lean implementation spreads from the boundaries of the stand-alone process to the entire organization, it produces suboptimal results or frustrating failures. Building on their research work Feng and Manuel (2008) stated that about 50% of operational-level implementations in hospitals have failed. A very similar result was reached by Glasgow *et al.* (2010), researchers reported that just over 60% of systemic implementation failed in a hospital setting. Authors investigating this issue argue that failures are due to the inability of organizations to deal with critical failure factors and to define effective paradigm introduction and diffusion strategies based on internal and external contextual factors (Coles *et al.*, 2017). While contextual factors refer either to the structural, managerial and cultural characteristics of organizations or exogenous environmental factors in the state before the Lean introduction, critical failure factors refer to organizational barriers during the Lean dissemination phase (Coles *et al.*, 2017; McDermott *et al.*, 2022a). The failure factors related to the internal Lean context and those factors most critical to consider are not managing benchmarking activities (van Rossum *et al.*, 2016), thinking in “silos” in administrative and clinical areas (de Souza and Pidd, 2011), the lack of confidence in applying Lean methodology (McDermott *et al.*, 2022a), the absence or ineffectiveness of communication systems (Al-Balushi *et al.*, 2014; de Souza and Pidd, 2011), the inability to manage cross-functional teams (Bhat *et al.*, 2019; McDermott *et al.*, 2022a), and, most importantly, the lack of a project management approach and skillset (Henrique *et al.*, 2021). Healthcare organizations, especially public ones, frequently ignore or deal reactively with changes in the external environment, i.e. public health policies, new managerial best practices, major challenges and opportunities arising from epidemiological transition and new technologies (Rosa *et al.*, 2021; Feng and Manuel, 2008). While the failure to manage internal contextual factors results in the inability to define an effective Lean implementation strategy, the failure to deal with external factors results in a great effort to adapt to the environment, in turn affecting the organization's readiness to focus on the Lean paradigm (van Rossum *et al.*, 2016). A successful implementation of Lean depends on an organization's ability to understand its stakeholder value and optimize its processes for its customers. Understanding patient and stakeholder perspectives is key to designing an effective implementation strategy (Womack *et al.*, 1990). In relation to critical failure factors for Lean deployment, the cited factors are a lack of financial investment in training and education for Lean; not enough time allocated for training and working on projects; a lack of managerial support and staff commitment; lack of leadership support; poor communication systems; no alignment of the continuous improvement program with strategy, aims and goals of Lean projects (Marolla *et al.*, 2022). Other failure factors which can be difficult barriers to overcome organizationally during Lean deployment can be a lack of project management skills, an unclear Lean implementation roadmap, poor data collection systems, a lack of project performance measurement systems and a lack of Lean practitioners (McDermott *et al.*, 2022a; Radnor *et al.*, 2011). Some studies have highlighted that government regional or national health departments or agencies can supporting health organizations to successfully implement Lean (Robert *et al.*, 2020). They argue that through policies and programs designed to incentivize and facilitate the implementation of the paradigm, agencies can stimulate its adoption by healthcare organizations. These programs can take different forms: funding for consulting and training activities, training events, conferences, etc (Robert *et al.*, 2020). The Productive Ward program draws on principles of Lean thinking and is an example of an initiative moved to disseminate the Lean paradigm in English hospitals (Smith and Rudd, 2010; Robert *et al.*, 2020). The program was developed through a collaborative design process involving the National Health Service Institute for Innovation (NHSI), national nurse leaders and industry partners (Robert *et al.*, 2020). National Health Service (NHS) testing sites and Learning Partners piloted the initiative and disseminated it nationally. Among the most significant research exploring this issue is Glasgow *et al.*’s (Dannapfel *et al.*, 2014) research. By analysing the Östergötland county council (COÖ) dissemination strategy and comparing it to the benchmark case dissemination strategies of NHSI and improvement in Great Britain and Odense University Hospital in Denmark, the authors identified common initiatives to foster successful dissemination and adoption of the paradigm by healthcare organizations. Although their research provided policymakers with interesting insights regarding key success factors for implementing a Lean dissemination strategy program, it does not evaluate whether the COÖ strategy was successful. Researchers have not investigated participants' lived experiences of agency-sponsored programs, so it is impossible to understand their effectiveness in perceived quality, level of Lean adoption, and the program's ability to foster implementation over time. The main success factors of multi-organizational Lean deployment initiatives undertaken by agencies include stakeholder involvement in program development; long-term strategic planning; effective communication with stakeholders to raise awareness of continuous improvement in healthcare; the ability to motivate organizations to embark on the implementation journey; defining a clear vision of objectives; developing a training and implementation support program; and testing and improving the program over time.

## 3. Case description

### 3.1 Background

To better contextualize the research methodology and the results obtained, this section discusses the role of the AReSS, the objective of the “Lean Lab” program and its implementation framework.

The AReSS was established by Italian Regional Law No. 29 on July 24, 2017. It is a technical-operational agency established to support the definition and management of social and health policies of the Apulia Region in Italy. The mission of AReSS is to propose, organize and improve the readiness of the regional healthcare system's response to the needs and expectations of stakeholder healthcare demand. To this end, the agency continuously monitors and assesses regional healthcare organizations' quality and cost performance and defines, plans and promotes development lines in the health and social welfare areas (AReSS, 2023). In addition, AReSS implements and promotes strategies focused on health and social service innovation and aimed at fully satisfying healthcare needs. It acquires and develops new strategic and organizational knowledge as a strategic agency. To this end, it experiments with paths of innovation and improvement, analyses, and disseminates existing national and international best health care practices, and promotes innovative clinical governance management models in compliance with the requirements of rationalization and optimization of spending from the regional budget.

### 3.2 Apulian healthcare pilot

**Figure 1:**
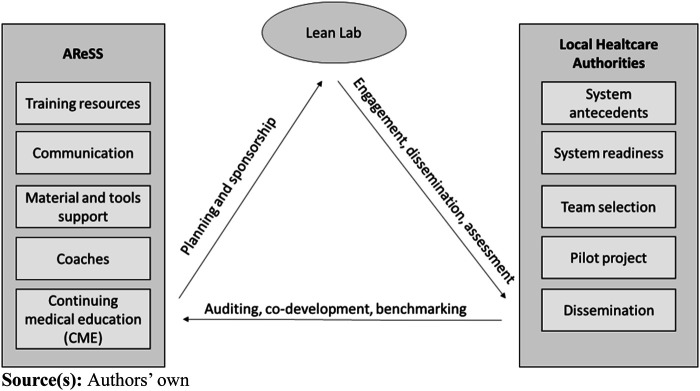
Framework describing the “Lean Lab” program

In 2018, AReSS, following benchmarking based on the National Outcomes Plan (Programma Nazionale Esiti (the national monitoring system for clinical performance of healthcare providers) and the clinical-management best practices in place, highlighted the need for Apulia's healthcare providers to increase the quality of patient pathways and care services. The agency identified Lean as an enabler for aiding their strategic goals while reducing waste and improving working situations in Apulian healthcare organizations. Apulia, also known by its Italian name Puglia is a southern region forming the heel of Italy's “boot.” AReSS thus proposed to the Apulian local healthcare authorities or Unità Sanitaria Locale's (USLs) that all ten of them collaborate to design a strategic plan for developing and implementing a Lean initiative at the regional level. The USL is the set of facilities, offices and services organized in each geographical area through which the different municipalities provide citizens with health care per the principles and objectives of the Italian National Health Service. Within its responsibility, the USL performs various tasks of prevention, diagnosis, treatment, rehabilitation, and forensic medicine. In 2018, the program was developed through collaboration between the AReSS, and a scientific committee composed of senior managers from the Apulian USLs and representatives of physicians and nurses from those organizations. The program framework was based on “The Productive Ward” model implemented by the NHS (Smith and Rudd, 2010) and was developed considering the contextual factors of Apulian healthcare policies. At the end of 2018, the program was defined in detail and was named “Lean Lab” (Figure 1).

**Figure 2:**
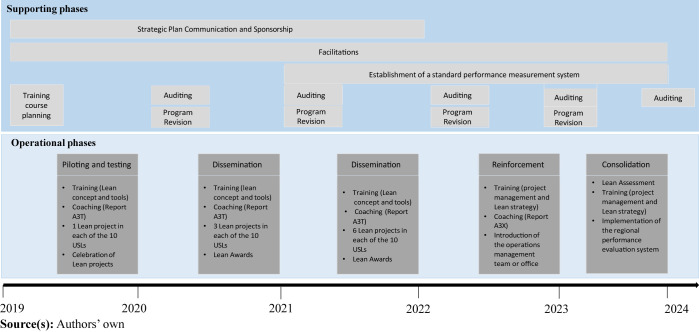
Representation of “Lean Lab” program phases

The 5-year strategic program consists of one-year operational and support phases. The operational phases are piloting and testing, dissemination, reinforcement, and consolidation. The support includes communication of the strategic plan, program revisions, auditing, facilitation, and establishment of regional standards performance measurement system (Figure 2). While the operational phases are focused on training and micro and meso implementation and involve the active participation of physicians, nurses and administrative staff belonging to healthcare organizations, the support phases are aimed at sponsoring the program, improving it, monitoring its effectiveness over time, and establishing effective regional healthcare performance measurement system. The support phase is conducted by AReSS staff with the assistance of USL staff trained during the program. Figure 2 shows the program timeline. Except for the phases of strategic communication, facilitation, creation of the regional standard performance measurement system and consolidation, all other phases run for six months.

### 3.3 Commencing the pilot

The start-up phases include the sponsorship campaign, recruitment of participants from USLs, and planning training and coaching activities. AReSS requested each of the ten Apulian USLs to select one multidisciplinary group composed of physicians, nurses, and administrative officers (5–6 professionals) from among the candidates willing to participate in the piloting and testing phase. In the next dissemination phases, each USL must select three multidisciplinary teams of 5–6 members from different organizations. The selection calls for group participants to belong to the process on which the Lean improvement project is to be evaluated. The project should be selected based on the group members' skills and the process's strategic importance. At least three groups from each USL and one executive from each healthcare organization participate in the reinforcement and consolidation phases. Groups must be selected from those who participated in the previous operational phases. While the main purpose of the first three operational phases is to introduce Lean and trigger micro-implementation, the other two operational phases aim to promote meso-implementation in organizations. Each operational phase comprises three sub-phases: training, coaching and project execution. Moreover, the piloting and testing phase includes a celebration sub-phase, while the dissemination phase includes a Lean Award sub-phase. At the end of each operational phase, before the activation of the next, there is a phase of upgrading and reviewing the scheduled activities. These adjustments are decided based on participant feedback and the results obtained (auditing phase). The training, coaching and project implementation modalities of the first three operational phases are similar. They involve a 60-h training course divided into a 40-h and a 20-h module, 15 h of coaching per project implemented, and two project moments. During the first 4 weeks, the first 40 h of training are conducted in the presence of all participants. The first training package consists of 5 h dedicated to testimonies of projects implemented in healthcare organizations outside the region, 25 h to explain the basic concepts and tools of Lean and to provide instructions for conducting an improvement project using the A3T Report (the tool summarizing the roadmap of the Deming Plan-Do-Check-Act cycle), and finally, the last 10 h are dedicated to serious games and exercises. Experienced Lean clinicians from other regions lead the testimonies showing projects implemented with Lean tools and A3T Reports, while external Lean consultants conduct the exercises. At the end of the first 40 h of training (4 weeks of 10 h), participants are required to initiate an improvement project (Plan and Do phases) on a patient pathway of their own. During the three months planned to conduct these phases, each team is provided 10 h of coaching by a Lean expert selected by AReSS. Coaches are responsible for monitoring the status of the project's progress. At the end of the Plan and Do phases, there is a 5-h inter-team discussion session followed by two days of training (10 h per day) to explain the methodologies and tools for conducting the Check and Act phases of the Deming cycle. These sessions take place with all participants attending. Over two months, the teams are supported by coaches (5 h per group) in conducting follow-ups and project improvements. A 5-h final meeting is planned to present and celebrate the projects and discuss the experience of each group. At the end of an operational phase, participants participate in sponsoring and communicating the “Lean Lab” program within healthcare organizations by playing the role of dissemination practitioners. They are requested to provide peer-to-peer training to introduce Lean to colleagues and encourage them to embark on a Lean project. In the month before the dissemination phases, dissemination practitioners receive 20 h of training on kata coaching. Finally, dissemination practitioners play the role of coaches and testimonies in the dissemination, reinforcement, and consolidation phases. Among the operational sessions, as part of the communication program, AReSS plans multiple sessions to present and celebrate projects conducted at the regional level. In addition, the agency sponsors the publication of the best projects conducted and the participation of dissemination practitioners in national and international conferences. Through the Lean Award sub-phase, AReSS aims to promote the culture of continuous improvement and to enhance the organizations' ability to benchmark. The reinforcement phase includes 50 h of managerial and operational training on project management and 15 h of coaching to implement Hoshin Kanri (A3X Report), a tool for the strategic management of Lean projects. Training hours are administered during the first month of the reinforcement phase, while Hoshin Kanri implementation activities are conducted in the second month. AReSS wants to raise awareness among organizations to formally establish a resolute team or office to monitor and govern micro and meso Lean implementation initiatives through this operational phase. Finally, the consolidation phase is geared towards improving the acquisition of project management skills to assess organizational maturity in exploiting the paradigm and establishing a regional monitoring system of organizational performance. The training activities of the consolidation phase and the implementation methods of the standard regional performance measurement system have not yet been fully defined. As written above, each planned phase can be adapted over time due to stakeholder feedback. In the “Lean Lab” presentation report, AReSS stated that the program was designed to be as inclusive and participant oriented as possible.

## 4. Methodology

### 4.1 Study setting and design

Based on the research by Dannapfel *et al.* (2014), this study aims to investigate the impact of the “Lean Lab” – the AReSS strategic Lean dissemination program – on adopting Lean in Apulian healthcare organizations. However, in contrast to the research by Dannapfel *et al.* (2014), the research focuses on the real-time and “lived” experiences of the pilot project participants of the organizations involved. In addition, the research covers three “Lean Lab” operative phases over almost four years.

The first phase was Lean introduction (2019), while the second and third related to Lean dissemination phases in Apulian healthcare organizations (2020–2021, 2021–22). As the “Lean Lab” is a 5-year strategic program implemented in January 2019, the case study's results cover the entire time span of the program. The methodology was qualitative, and multiple data sources and collection methods were used. Using triangulation methodology (Yin, 2016), data is analysed by the researchers. The data sources utilized are AReSS documents related to the design and implementation of the “Lean Lab” program, reports from healthcare organizations, presentations of implemented Lean projects, direct observation (more than 100 h) and two semi-structured interviews with piloting and testing phases participants – also referred to as dissemination practitioners (30 min per panel member per interview). Of the study authors involved in the conception and design of the program, one was also a Lean trainer and an operational coach during the program.

### 4.2 Interview timelines

The first author conducted the interviews and recorded the responses. These were analysed with the assistance of the first and third authors. Interviews were administered to each of the fifty-two participants in the first operational phase of the program in December 2019 and May 2022. The first interview focused on the early experiences of introducing Lean through a pilot project, and the second interview was on the dissemination phase in organizations they belong to. During the second operative phase of the program, the fifty-two participants were named as disseminator practitioners as their roles changed from learners and deployers of the pilot projects into trainers, Lean leaders, and Lean coaches of the subsequent projects implemented in healthcare organizations. The participants are grouped into nine groups of five and one group of seven, each from one of the ten USLs in the Apulia region.

### 4.3 Conducting the interviews

The interviews were structured to reveal participants' experiences according to the determinants of Lean implementation success proposed by the Model for Understanding Success in Quality (Kaplan *et al.*, 2012). The macro-factors investigated are the external environment, organization, quality improvement team and micro-system. Questions were a mix of structured and unstructured in order to aid the interviewees in elaborating and expressing their opinions (Bellotti, 2014). Some interviews were face to face and others were held on Microsoft Teams depending on the interviewee preference.

### 4.4 Thematic analysis

All transcripts were uploaded to Microsoft Excel so thematic analysis could be facilitated (Alhojailan, 2012). Thematic analysis was conducted and coding of themes by the research theme. Coding and memoing aided the organization of the themes and subthemes (Birks *et al.*, 2008; Cascio *et al.*, 2019). Inter-rater reliability testing was carried out to ensure elimination of bias and consensus (Fleiss *et al.*, 2003). A value of 90% (0.9) was calculated utilizing Cohens Kappa to ensure inter-rater reliability (Goodwin, 2001).

## 5. Results

The first set of results discusses the participants' experiences of the operational phases of Lean piloting and testing, and dissemination in various subsections and their perceptions regarding the support given to the Lean program. A second set of interviews was conducted in mid-late 2022 before the activation of the reinforcement phase. Finally, from January 2022 to May 2022, the dissemination practitioners functioned as Lean disseminators within their organizations and participated in the auditing and review phases of the Lean Lab. The results are aggregated per group of participants. Each group is assigned a label G_x_ where G represents an individual group and X ranges from 1 to 10.

### 5.1 Piloting and testing phase (Jan 2019–Dec 2019)

Initially, one hundred applicants who applied for the Lean piloting and testing program were received (distributed equally among the USLs). To gain trust in the Lean Lab program and overcome mistrust, a good communication program was important (G_1–10_). Many participants were apprehensive about participating in the “Lean Lab”, having experienced past failures in implementing improvement programs. The communication campaign included information in relation to the context of Lean in healthcare as well as examples of Lean applied externally in terms of benchmarking activities and the presentation of healthcare policies aimed at improving waste and optimizing stakeholder value (G_1–10_). The opportunity to participate in a long-term strategic Lean program which was to be sponsored by the directorates, allowed learning about Lean applications in other regions, which represents further motivating factors for the participants to get involved (G_1–3_, _6, 8–10_). G_2–4, 6–10_ stressed that the initial communication campaign encouraged them to consider getting involved in the Lean program. Before the communication sessions, participants viewed Lean as a tool oriented exclusively to reducing costs and increasing productivity at the expense of occupational well-being. G_1–2, 9–10_ emphasized the importance of communication of the Lean implementation program in terms of the operational and strategic objectives of the program. All participants in the Lean Lab design committee (11 from 10 USLs) applied for and were selected to participate in the program. The explanations of how the program would run in terms of the piloting and testing phase were appreciated by all groups. The participant testimonials that demonstrated increased confidence in the methodology and the Lean serious games that created a strong team working environment were of primary importance for participant motivation purposes during the training sub-phase. The training hours in Lean theory were also considered critical for providing a foundation for understanding the methodology and introducing the Lean tools. An Example of some of the elements of the training and implementation phase which the participants found positive was outlined in Table 1.

All groups completed the project implementation sub-phase. All the hospitals had a suite of improvement projects related to patient-facing process pathways. The objective of each project was to increase the value-add increase for patients. At the end of the review phase, most projects had returned higher results than expected (G_1–7, 10_). Project goal setting and progress reviews were conducted by each team with the Lean project coach. Goals were set to be clear, challenging, and measurable (G_1–10_). Some examples are project results are demonstrated in Table 2.

#### 5.1.1 Challenges and success factors during the project implementation sub-phase

The groups described the project implementation sub-phase as interesting and challenging, which all participants embraced with high motivation and enthusiasm. The Lean coaches were a key critical success factor for the micro-implementation as they supported the Lean team members during the activities considered the most complex and difficult in applying the tools in analysing the as-is state of the process (G_1–7; 9–10_).

All participants reiterated the difficulty of data collection in patient-facing processes such as adopting data collection methods and tools, completing Gemba Walks and using value stream mapping tools (G_1–10_).

G_4, 5, 8,__10_ stated that they found working on the project stressful regarding the time required to be spent on them and were incredibly stressed regarding the time they spent on it. Most project work took place outside of working hours and was unpaid.

G_1, 2, 6, 9_ reported that having managers who provided material resources for projects, e.g. facilities, whiteboards, and managerial support, e.g. data availability and data analysis, was crucial to the success of their project.

The Lean coaches enabled a culture of communication and trust by implementing norms of behaviour and rules in the group (G_1–3, 6, 8–10_). Collaborating on tasks and meeting deadlines created a feeling of mutual trust and respect among team members, enhancing the individual's motivation. G_1,3,6–7_ emphasized the importance of consensus-based decision-making based on factual data from process data analysis, which was not affected by cognitive bias.

Finally, all groups reported that they achieved more than they expected and understood the importance of proactively finding and addressing endemic problems instead of dealing with them reactively (G_1–10_). The project closing meetings and recognition phases were particularly acknowledged by all the groups (G_1–10_). The project closing meetings enabled an effective exchange of ideas among the groups (G_1–6; 8_), while the recognition phase further motivated participants through prestige-based reward systems (G_2–7; 9–10_).

### 5.2 Dissemination phases (Jan 2020–May 2022)

In January 2020, the members of the ten groups that participated in the first operational phase were appointed as dissemination practitioners and have been involved in the program communication activities and the program review and improvement phase. In addition, over the next 5-month period, the directors of the USLs deployed Lean dissemination practitioners to deliver peer training courses and to demonstrate the success of the projects implemented within their organizations. Although many Lean dissemination practitioners commended the program, they bemoaned the lack of time to deliver the training activities (G_1–7; 9–10_). Another issue was the absence of a formal vision and strategy for the directorates and ensuring voluntary course participation (G_2–5; 7; 8; 10_). The reward and recognition phase of the prior year's projects and the presentation of a Lean Award were the highest motivators for ongoing participation in the program (G_1–7; 10_). The sense of reward and, in many cases, of challenge and opportunity was pervasive (G_1–4; 5–7, 10_).

Success stories reported by dissemination practitioners are another factor that motivated many peers to attend the training courses and to participate in the dissemination phase (G_1, 4–7; 9–10_). All other motivational factors related to participation in the first operational phase were reiterated by participants in this phase (G_1–10_). In May 2020, the number of applications to participate in the Lean program exceeded 600. The directors of each USL selected medical personnel such as doctors and nurses from departments other than those of the participants involved in the piloting and testing phase. Each USL involved three groups in the dissemination phase, totalling 153 participants. The selection process included feedback from the Lean dissemination practitioners. According to the Lean dissemination practitioners, the methodology spread naturally and spontaneously in departments due to the proximity of the participants in areas where Lean projects were implemented (G_2–7; 10_). The inclusion of other peers allowed the Lean dissemination practitioners to implement multiple Lean projects in their departments even before the start of the dissemination phase (G_1, 3, 5–7, 10_). While some of these projects, conducted with the kaizen method, have yielded satisfactory results, others have failed (G_1, 3, 5–7, 10_). Dissemination practitioners have consistently pointed out the lack of management involvement in implemented projects (G_1–3, 5, 7–9_). During the dissemination phase, dissemination practitioners played the role of coach and mentor. The experiences and opinions of new program participants in relation to the training courses were the same as the Lean dissemination coaches (G_1–10_). During the implementation sub-phase of the project, each group was assisted by at least one Lean coach from their own USL and one external coach. The maturity achieved in applying and using Lean tools was not always enough to allow the dissemination operators to effectively assist their colleagues during the improvement projects (G_1–10_). While internal coaches were skilled in basic mapping tools, they could not manage multidisciplinary teams or employ complex data analysis models. A Lean Kata coaching course administered before the dissemination phase was rated as highly positive by dissemination practitioners (G_1–10_).

As of December 2020, at the end of the project implementation sub-phase, 26 projects had achieved the planned results, while 4 had failed. Of these, twenty projects were successful and focused on patient value add, four focused on improving the value-add resource management of the organization and two on improving the quality of the working environment. The projects that failed were aimed at enhancing the integration of the individual hospitals and territory processes. Unsurprisingly, these more strategic projects failed as there was a lack of commitment and support from leadership and management, which was raised as a failure factor throughout the program by participants (G_1,3,6,7_). At the end of the first dissemination phase, the Lean dissemination practitioners highlighted several problems in relation to the program.

Many participants reiterated the lack of time they were allocated to devote to projects (G_1–7,10_); although the directorates showed great lip service to them, they often did not support or facilitate project implementation (G_1–3, 5,__7–10_) and kata coaching requires too much time (G_1–10_). The dissemination practitioners proposed to include time spent on coaching activities as the working time to overcome kata takes too much extra time (G_1–10_). This proposal was not accepted by all the directorates of the USLs (G_1–10_). Prior to the second phase of dissemination in all healthcare organizations, Lean concepts were starting to spread spontaneously (G_1, 3–7, 9–10_). Several USLs had activated an internal Lean Award to stimulate the implementation of Lean projects to avoid including overtime hours in pay (G_1, 6–8, 10_). Before May 2021, dissemination practitioners had reported that many projects were implemented without their knowledge and were arising spontaneously (G_2–5; 8_). Spontaneous Lean projects were implemented in clinical pathways. For example, in one USL, a Lean project was conducted to improve value and add administrative activities related to the booking of outpatient visits and the continuity of patient care (G_4_).

In 2021, Lean dissemination practitioners supported by participants in the first dissemination phase will be continued with training, coaching and communication activities (G_1–10_). For the second consecutive year, the number of applications to participate in the operational phase had increased: from about 600 to just over 800. By the same selection method as in the first dissemination phase, 6 groups were selected from each USL from a total of 312 participants. Perceptions of the training, project execution and Lean award sub-phases of the second dissemination session were the same as those from the first deployment session (G_1–10_). However, some Lean dissemination practitioners mentioned a lack in relation to the management of the dissemination process (G_3–8; 10_). As the number of projects was growing rapidly, the organization should have set up a dedicated program management office or structure to monitor and support the projects (G_3–8; 10_). These control rooms or project management offices needed to be composed of operational managers, doctors and nurses trained in Lean. This suggestion was also made prior to this reinforcement phase. Between January and May 2022, micro implementations increased in all USLs (G_1–10_) (Table 3). However, although the number of projects increased, the failure rate increased more than proportionally. Moreover, organizations were no longer able to govern the dissemination process.

In those USLs where the recognition and reward of the Lean Award were not introduced, many projects were implemented without ever being communicated to management (G_2–5_). The dissemination practitioners highlighted that as the number of projects increased, there was conflict regarding priorities regarding time allocated to working on projects and resource allocation (G_1–7, 10_). Also, there were examples in which the improvements in the performance of certain patient process pathways (in terms of execution time, waiting time, a saturation of resource capacity utilization and quality as perceived by patients) were counterbalanced by a reduction in the performance of other processes that shared the same resources (G_1, 5–7, 10_). Although dissemination grew naturally and maturity in applying Lean tools increased, organizations could not govern implementation at the meso level (G_1–10_). The lack of a dedicated framework to drive Lean dissemination and management involvement and deliver clear, formalized strategies are considered the main barriers to meso implementation (G_1–6, 8–10_).

Before the operational phase of the deployment, dissemination practitioners discussed with management setting up pilot project control rooms to support and monitor the spread of Lean in the different hospitals (G_1–4, 6, 8–10_). The directorates had planned to pilot this recommendation with AReSS during the “Lean Lab” program planning but were hesitant. Their concern was that assigning resources to control and monitor the dissemination of the Lean program might negatively impact organizational performance and generate internal conflicts (G_3–4, 6, 8–10_). In addition, the perception of dissemination practitioners was that the management team, having had no experience in project implementation, could not understand the need for this type of facilitation (G_1–4, 6, 8–10_). In May 2022, none of the USLs had a control room or had established an operational team manager (G_1–10_). Therefore, in the review phase (January – April 2020), dissemination practitioners stressed the criticality of primarily management staff participating in the reinforcement phase.

In April 2022, the publication of the National Outcome Plan System, covering the year 2020–2021, confirmed the improvements achieved in patient process pathways with Lean project implementation. One outstanding published study/project was one hospital's project to reduce the 30-day mortality rate of acute myocardial infarction (Rosa *et al.*, 2023b). The hospital had implemented a Lean project on this pathway as its performance was significantly under the national average: 12.3% compared to 7.8%. The performance reported by the National Outcomes Plan covering 2001 and 2002 showed 7.3 and 5.8% for that hospital (G_4_).

## 6. Discussion

The study answers the research questions. The success or failure of the regional Lean Lab program with a cross-organizational design and communication of the objectives and methods of implementation were among this program's most critical success factors (RQ1). The program's initial deployment succeeded in enhancing and sustaining the Lean program and the motivation to participate and eliminate barriers related to mistrust of improvement methodologies. The communication campaign was also important in spreading the potential of Lean and familiarizing potential participants with the concept of the value and the importance of external contextual factors. These results confirm what has been reported by Dannapfel *et al.* (2014) as well as in systematic literature reviews of Lean application in healthcare by Al-Balushi *et al.* (2014) and McDermott *et al.* (2022).

The training sub-phase, particularly through testimonials and serious games, further increased motivation and trust. Deploying adequate training is an important critical success factor in Lean deployment and building trust (McDermott *et al.*, 2022b). Furthermore, from an operational point of view, the training and coaching were instrumental in the Lean program introduction and dissemination phase at the micro level. The celebration and the Lean Award sub-stages were other elements of great value in the programs success (RQ2). These resulted in the rise of feelings of emulation and challenge among the participants during the operational phases of the program. Recognition and reward of Lean success are important for building a good culture and promoting teamwork (Al-Balushi *et al.*, 2014). While the assignment of witnessing, coaching and communication tasks to the dissemination practitioners during the operational phases allowed them to gain experience in both the use of tools and project management, the workload assigned was also negatively evaluated because it was not recognized as working time.

In summary, although the dissemination practitioners were willing to take on the assigned roles, the difficulties they experienced in relation to time availability were a major obstacle. However, it is crucial to remember that the role of the dissemination practitioners was decisive for dissemination. Trust in colleagues, the sense of belonging and the possibility of benefiting from an internal coach prompted many doctors and nurses to embrace the Lean change (McDermott *et al.*, 2022b). Furthermore, the dissemination practitioners' support and dissemination activities ensured the programs continuity over time (Bhat *et al.*, 2019). Even though the operational phases took place from May to December each year, organizations were always engaged in the micro-implementation and dissemination phase of Lean. This increased the speed of dissemination and increased employee involvement.

A lesson learned from the implementation experience within the organizations was that the celebration and success of pilot projects was key instigator of the dissemination phase (RQ3). However, it was demonstrated by the practitioners that dissemination happened more fluidly in units with at least one Lean coach, while it is slow or nonexistent where there is no staff training in Lean. Internal training is a critical success factor for motivating potential practitioners, disseminating a culture change and aiding understanding of Lean (McDermott *et al.*, 2022a; Trakulsunti *et al.*, 2021). Until improvement teams reach maturity levels, the presence of a Lean coach (internal or external to the organization) is another critical determining factor for micro-implementation initiatives. Some Lean tools, such as simulation and data modelling, cannot be easily applied to have use for application by doctors and nurses (McDermott *et al.*, 2022), requiring more experienced Lean coaches to complete more complex applications. Finally, dissemination practitioners deemed the managers' commitment a critical factor during the introduction and dissemination phases. Management demonstrated commitment by facilitating project sessions and having an internal coach.

Conversely, the lack of management commitment is a failure factor of both micro- and meso-implementation. Based on the study's findings, even though healthcare organizations have deployed resources and personnel in planning and implementing the introduction and dissemination phases of Lean, they have not been able to sustain and maintain the latter phases of the Lean projects to their maturity. While the initial Lean teamwork, culture, training, and projects were embraced and adapted, there was not a high level of strategic organization. As a result, the number of projects, failures, or sub-optimizations increased, as healthcare organizations could not adopt robust project management systems or consider implementing more stringent governance structures for monitoring and controlling the Lean. As with many Lean deployment failures, this was due to fears and a lack of strategic vision on the part of management (Antony *et al.*, 2021; McDermott *et al.*, 2022).

In conclusion, the regional “Lean Lab” plan strongly impacted the introduction and deployment of the Lean program methodology. The organizations empowered the staff, doctors, and nurses who participated in the program to lead the program's deployment. The participants' experiences were evaluated as positive in the first two years and until the complexity of the dissemination phase management was no longer under control. After two years, organizations can still not implement Lean on an operational or meso level despite many of their employees having reached a high maturity level in applying Lean tools. It is critical for successful meso implementation that leadership and management are strongly committed to and embraces Lean strategically (McDermott *et al.*, 2022; Rosa *et al.*, 2021). A central control room or a resolute team committed to Lean project management and dissemination and a governance process should be implemented to support management.

## 7. Conclusion

This study was one of the first long-term studies of a healthcare organizational Lean deployment across several hospitals and an entire healthcare region under one directorate. The study is unique in the size, spread and strategic nature of the deployment as well as the insights it provides into the knowledge and literature in relation to the critical factors in driving the success or failure of the intra-regional initiative. It is also unique in that it garners the participants' experiences in Lean introduction and dissemination in their organizations and in managing the sustainability of Lean implementation over time, which was a three-and-a-half-year period.

The study's limitations are that it is only a study across one regional healthcare organization despite including 10 different hospitals. Further research could expand the case study beyond four years and expand the case study into other healthcare regions both in Italy and globally.

The study has many theoretical and managerial implications. From a managerial point of view, this study provides valuable insights into Lean deployment in hospitals and across a regional hospital healthcare group. This study is the first of its kind at this strategic level implementation and is a longitudinal study of close to four years. The study provides unique insights into the progress of the Lean deployment over time and from the healthcare practitioner and participants' viewpoint, which can inform further healthcare organizational deployments of Lean. From a theoretical point of view, this study adds to the state-of-the-art literature in relation to Lean in healthcare, demonstrating how Lean can be deployed in a healthcare organization and the specific organizational differences to other sectors. Finally, this study can inform policy and structure around the setup and implementation of a successful Lean program in healthcare to implement a best practice model of Lean deployment.

Future research opportunities are to continue the case study over the next stages of implementation and evaluate the sustainability.

## Figures and Tables

**Table 1:** Elements of the training and implementation phases which participants favoured

(G2, 5, 7–8) claimed that through applying the Lean serious games and testimonies, their concerns regarding the difficulty of implementation were allayed
The participants highly valued the training and implementation phases of the Lean projects being divided into two phases (G2, 5,8–10)
The chance to define the application areas of the project with experts and managers (G_2–4; 7–8, 10_)
The time allocated to run the Lean project (G_4–6, 10_)

**Source(s):** Authors' own

**Table 2:** Examples of project results

Increase in the percentage of elderly patients getting surgery for hip fracture within 48 h from 59 to 75% (follow-up measured performance: 82%)
Reduction in average yellow code waiting times in first-aid units from 41 to 32 min (follow-up measured performance: 28 min)
Lead time reduction of the oncology patient pathways undergoing mid and long-term chemotherapy from 152 and 241 min to 130 and 200 min, respectively (follow-up measured performance: 127 and 194 min)

**Source(s):** Authors' own

**Table 3:** Lean dissemination in Apulian USLs

	Year	Internal coach	Internal training hours	Number of internal training courses	Staff trained by in-house training. Courses	Lean project detected	Success	Fail	Internal Lean award (Y/N)	Dedicated structure to monitor and support the projects (Y/N)**
USL1	2019	0	0	0	0	1	1		N	N
	2020	5	30	2	11	9	8	1	N	N
	2021	21	30	2	18	11	8	3	Y	N
	2022*	51	30	2	24	6	4	2	Y	N
USL1	2019	0	0	0	0	1	1		Y	N
	2020	5	40	2	21	5	4	1	N	N
	2021	20	40	2	25	8	8		N	N
	2022*	50	40	2	26	6	4	2	N	N
USL2	2019	0	0	0	0	1	1		N	N
	2020	5	20	1	15	8	8		N	N
	2021	21	32	1	10	9	6	4	N	N
	2022*	51	32	1	12	6	5	1	N	N
USL3	2019	0	0	0	0	1	1		N	N
	2020	5	30	2	14	5	5		N	N
	2021	21	36	2	16	9	6	3	N	N
	2022*	51	36	2	18	6	5	1	N	N
USL4	2019	0	0	0	0	1	1		N	N
	2020	5	32	2	17	5	4	1	N	N
	2021	20	36	2	25	7	5	2	N	N
	2022*	50	36	2	24	9	7	2	N	N
USL5	2019	0	0	0	0	1	1		N	N
	2020	5	20	1	15	4	4		N	N
	2021	20	20	1	11	6	6		N	N
	2022*	50	32	2	18	8	7	1	N	N
USL6	2019	0	0	0	0	1	1		N	N
	2020	7	36	2	31	7	7		Y	N
	2021	22	28	2	33	11	9	2	Y	N
	2022*	52	28	2	31	5	5		Y	N
USL7	2019	0	0	0	0	1	1		N	N
	2020	5	40	2	22	5	5		N	N
	2021	20	40	2	20	8	7	1	N	N
	2022*	50	40	2	32	10	8	2	N	N
USL8	2019	0	0	0	0	1	1		N	N
	2020	5	16	1	18	6	6		Y	N
	2021	20	16	1	24	10	8	2	Y	N
	2022*	50	16	1	14	8	6	2	Y	N
USL9	2019	0	0	0	0	1	1		N	N
	2020	5	32	2	32	7	7		N	N
	2021	20	60	4	54	8	7	1	N	N
	2022*	50	60	4	60	11	7	4	N	N
USL10	2019	0	0	0	0	1	1		N	N
	2020	5	32	2	25	8	8		Y	N
	2021	20	32	2	31	12	10	2	Y	N
	2022*	50	32	2	25	9	6	3	Y	N

**Note(s):** *Period: January – May 2022

**In some USLs, the number of projects implemented may differ from those shown in the table

**Source(s):** Authors' own
